# The Reliability and Validity of the Center for Epidemiologic Studies Depression Scale (CES-D) for Chinese University Students

**DOI:** 10.3389/fpsyt.2019.00315

**Published:** 2019-05-21

**Authors:** Lijun Jiang, Ying Wang, Yining Zhang, Rui Li, Huailiang Wu, Chenyi Li, Yunlin Wu, Qian Tao

**Affiliations:** ^1^Department of Public Health and Preventive Medicine, School of Basic Medicine, Jinan University, Guangzhou, China; ^2^Division of Medical Psychology and Behavior Science, School of Basic Medicine, Jinan University, Guangzhou, China; ^3^Medical Imaging Center, First Affiliated Hospital of Jinan University, Guangzhou, China; ^4^International School, Jinan University, Guangzhou, China; ^5^Center for Brain Science and Brain-Inspired Intelligence, Guangdong–Hong Kong–Macao Greater Bay Area, Guangzhou, China

**Keywords:** Center for Epidemiologic Studies Depression Scale, reliability, validity, students, depression

## Abstract

**Aims:** Depression is prevalent among university students worldwide, and the prevalence appears to be increasing. As an intermediate stage between being healthy and having depression, students with subthreshold depression could develop worsening depression or recover with intervention to prevent depression. The Center for Epidemiologic Studies Depression Scale (CES-D) is a useful tool to assess subthreshold depression. The primary purpose of the current study was to evaluate the psychometric characteristics of CES-D in Chinese university students. Secondly, we aimed to describe the prevalence of subthreshold depression among the student sample and examine its demographic correlates.

**Methods:** A total of 2,068 university students participated in the study, and they were asked to respond to the Chinese CES-D, Beck Depression Inventory-II (BDI-II), and Positive and Negative Affect Schedule (PANAS). The factor structure was evaluated by conducting exploratory (EFA) and confirmatory factor analysis (CFA) using a structural equation modeling approach. The reliability was assessed by calculating Cronbach’s alpha, inter-item correlation, and item-total correlation coefficients. The prevalence of subthreshold depression was calculated and demographic correlates of gender, grade, and major were examined by multiple regression.

**Results:** The final sample included 1,920 participants. The EFA results suggested extraction of three factors (somatic symptoms, negative affect, and anhedonia) that account for 52.68% of total variance. The CFA results suggested that the newly derived model with 14 items was the best fit for our data. Six items were removed from the original scale (item 9, 10, 13, 15, 17, and 19). The Cronbach’s alpha of the 14-item CES-D was 0.87. The prevalence of subthreshold depression among university students reached 32.7% for the 20-item CES-D and 31% for the 14-item CES-D, although there was no significant difference of prevalence in gender, grade, and major.

**Conclusions:** The CES-D has good reliability and validity for assessing subthreshold depression in Chinese university students.

## Introduction

Depression is a common but serious mental illness typically characterized by sad, hopeless, or anxious feelings. The age of onset for depression has been falling, making university students particularly vulnerable to developing depression (1). In addition, university can be a challenging time for students, as students struggle with leaving home for the first time, living independently, increasing academic pressures, forming new relationships, and making important decisions. A growing body of evidence suggests that depression is prevalent among university students worldwide (2, 3), and this prevalence appears to be increasing. Untreated depression can persist for a long period, which may interfere with students’ daily lives, including academic performance and social functioning (4). In severe cases, depression may induce substance abuse (5, 6) and suicide (7). Recognition of the warning signs and early diagnosis of depression are, therefore, crucial for treating depressive symptoms and preventing depression from returning.

A prodromal phase of depression is regarded as subthreshold depression, in which depressive symptoms do not meet the criteria for a major depressive disorder (8). As an intermediate stage between being healthy and having depression, individuals with subthreshold depression could be worsened and develop depression (9) or recover with intervention and finally depression could be prevented (10). A useful tool to assess subthreshold depression is the Center for Epidemiologic Studies Depression Scale (CES-D), which was designed for use in epidemiology studies to assess degrees of depressive symptoms and detect at-risk individuals for depression in the general population (11). The CES-D is a self-rating 20-item scale with a recommended threshold score of 16 for indicating the presence of subthreshold depression. The current literature on CES-D has reported at least 20 factor solutions in different populations and subpopulations. Several items were questioned on their validity and psychometric properties. For instance, item 17 (“I had crying spells”) is biased by gender, as suggested by the differential item functioning analyses (12–14). The two items (item 15 “People were unfriendly” and item 19 “I felt that people dislike me”) measure interpersonal problems, which are not consistent with theories of depression and widely used diagnosis criteria for depression (15, 16). The CES-D seems to be the only test that includes an interpersonal factor, but the other widely used instruments, such as the Beck Depression Inventory (BDI), the Hamilton Rating Scale for Depression (HRSD), and the Zung Self-rating Depression Scale (SDS) (16) do not have such a factor. The CES-D has demonstrated good reliability and validity across various Chinese populations, such as those who attempt suicide (17), patients with type 2 diabetes (18), primary care patients (19), and the elderly community (20). It is important to examine the reliability and validity of CES-D Chinese version in university students in order to advance detection and intervention of subthreshold depression.

The primary purpose of the current study was to evaluate the psychometric characteristics of CES-D in Chinese university students. Secondly, we aimed to describe the prevalence of subthreshold depression among the student sample and examine its demographic correlates.

## Methods

### Participants and Procedure

The study was conducted in Guangzhou in Southeastern China. There are more than ten universities in Guangzhou, but only five of them are comprehensive universities that include a variety of majors, such as literature and management, science and engineering, and medicine. Among the five comprehensive universities, two are national key universities and the other three are ordinary universities. In order to obtain a representative sample, we randomly selected one from two national key universities and one from three ordinary universities. We recruited students from one national key university (Jinan University) and one ordinary university (Guangzhou University) as the study participants. A stratified cluster selection strategy was used to recruit the participants. We stratified the sample into three majors: literature and management, science and engineering, and medicine. Five classes of each major were randomly selected during the 2016/2017 academic year, and all students from the selected classes were invited to participate in the study. We personally contacted the students and invited them to participate in their respective classrooms after the end of a class. Several studies raised problems associated with small samples in factor analysis and suggested large sample size. For instance, the effect of sample size on the results of factor analysis was empirically tested and the authors reported that larger samples tend to produce more accurate solutions (21). As the sample size increases, sampling error is reduced, factor analysis solutions become more stable and more reliably produce the factorial structure of the population (22). Given that the recommendations regarding sample size for factor analysis, a large sample size is expected (23). In total, 2,068 students were recruited in the study as participants.

### Instruments

#### The Center for Epidemiologic Studies Depression Scale

The CES-D scale was developed to screen for depression by measuring the frequency of events and ideas over the past week (11). The CES-D scale is a 20-item instrument with each item rated on a four-point scale ranging from 0 (“rarely or none of the time”) to 3 (“most or all of the time”). Four of the items are positive statements which are inversely scored for calculating the total score. The total score ranges from 0 to 60 and a higher score indicates a greater risk of depression. For the original CES-D scale, a total score of 16 or greater is considered as indicative of subthreshold depression (11). However, a number of studies have evaluated the diagnostic accuracy of the CES-D to detect depression at the general population and proposed a variety of cut-off scores, such as a cut-off score of 18 among a very old population living in residential homes (24), a cut-off score of 21 among type 2 diabetes and primary care patients (18), and a cut-off score of 22 among elders (25). Using a meta-analytic approach, a previous study systematically reviewed 28 CES-D studies and proposed an optimal cut-off score of 20 with sensitivity of 0.83, specificity of 0.78, and diagnostic odds ratio of 16.64 (26). The present study adopted a cut-off score of 20 for detecting subthreshold depression. The CES-D had been validated in a variety of Chinese samples. For instance, good reliability was demonstrated in suicide attempters and residents with Cronbach’s alpha values of 0.940 and 0.895, and a three-factor with 14 items was the best fit (17). Similarly, a sample of 3,686 primary care patients demonstrated good internal consistency (ခωH = 0.855) and good test–retest reliability (ICC = 0.91), and a bi-factor structure with 20 items was the best fit (19). The previous Chinese version (19, 27) was used and it was verified by back-translation (**Supplementary Material**).

#### The Beck Depression Inventory-II

The BDI-II was developed to screen for depression and it has been widely used to measure the severity of depression (28). The scale consists of 21 items, and each item is rated on a four-point scale ranging from 0 (“I do not feel sad”) to 3 (“I am so sad or unhappy that I can’t stand it”). The subscales of BDI-II consisted of somatic-affective (item 15, 16, 18–20) and cognitive factor (item 1–14, 17, 21) for undergraduate students (29). The total score ranges from 0 to 63, and a higher score suggests more severe depression. The severity of depression can be categorized into minimal depression (score 0 to 13), mild depression (score 14 to 19), moderate depression (score 20 to 28), and severe depression (score 29 to 63) (28). The Chinese version of BDI-II (30) had been validated in university students in mainland China (31) and Taiwan (32) with Cronbach’s alphas of 0.85 and 0.88, respectively.

#### The Positive and Negative Affect Schedule

The Positive and Negative Affect Schedule (PANAS) has been widely used to measure both positive and negative affect (33). The questionnaire contains two 10-item scales, and each item is rated on a five-point scale ranging from 1 (“very slightly or not at all”) to 5 (“very much”). The total score ranges from 10 to 50 for both positive and negative affect, and a higher score indicates a higher positive emotion and a higher negative emotion, respectively. The Chinese version of PANAS (34) had been validated in residents from community, with Cronbach’s alpha for positive and negative affect of 0.85 and 0.83, respectively.

### Data Analyses

The potential gender bias of item 17 was evaluated by estimating the differential item functioning using an item response theory approach (12). We produced the non-parametric item characteristic curves that were smoothed with a Gaussian kernel using jMetrik 4.1.1. An exploratory factor analysis (EFA) was followed by a confirmatory factor analysis (CFA) by splitting the data set into halves. We performed an EFA with the first half (Sample One) and then used the results to fit a CFA model to the second half of the data (Sample Two). EFA was performed using principal component analysis and oblique promax rotation. According to the statistics literature, a factor loading of 0.5 is used as the cut-off score for the most accepted norm of EFA (23, 35). CFA was performed using the weighted least squares with mean and variance adjustment (WLSMV) estimator (36, 37). All CFA models were estimated using Mplus 7.4 software, and the loading of the first indicator in each factor is automatically fixed to be 1.0. The model derived by EFA and five recommended models (11, 12, 18, 38, 39) were evaluated by CFA with and without the three items (item 15, 17, and 19). Multiple indices for fitness were used: root mean squared error of approximation (RMSEA) must be less than 0.08, with 90% confidence interval values below 0.10, Tucker–Lewis index (TLI) and comparative fit index (CFI) must be greater than 0.90 (40), and change in chi-square given the change in degrees of freedom should be less than 5.0. The Akaike information criterion (AIC) and Bayesian information criterion (BIC) were used to compare the non-nested competing models. Based on a structural equation modeling (SEM) approach, we calculated average variance extracted (AVE) and composite reliability (CR) (41, 42). Correlation coefficients between subscale scores of CES-D, BDI-II, and PANAS were calculated. To investigate the relationships between the underlying constructs of the CES-D and BDI-II, we built a two-factor measurement model to explore the latent structure of the CES-D and BDI-II in Sample Two (43), and this was checked by CFA. The reliability was evaluated by calculating Cronbach’s α, inter-item correlation, and corrected item-total correlation coefficients. The prevalence of subthreshold depression in the university student sample was calculated using a cut-off score of 20 for the 20-item CES-D (26). In addition, we performed receiver operator characteristic (ROC) analysis to determine the optimal cut-off score for the revised CES-D and calculated the prevalence. The relationship between the CES-D and demographic correlates was investigated by multiple regression.

### Ethics

This study was carried out in accordance with the recommendations of the Declaration of Helsinki with written informed consent from all subjects. The protocol was approved by the Ethics Committee of the School of Medical Science at Jinan University, China. For students with positive results, a notice indicating that they are at risk of subthreshold depression and developing depression was given. We further provided some guidance and suggestions, such as gaining access to university counseling service and talking to a friend or a family member.

## Results

### Demographic Characteristics

A total of 2,200 questionnaires were distributed, and 2,068 questionnaires were returned (94.00%). Invalid questionnaires with any questions unanswered were excluded and the final sample included 1,920 questionnaires (92.84%). Of these, 710 (36.98%) were male and 1,210 (63.02%) were female. There were 1,315 (68.49%) junior grade students (freshman and sophomore) and 605 (31.51%) senior grade students (junior, senior and fifth grade). Regarding the major, 592 (30.83%) literature and management, 619 (32.24%) science and engineering, and 709 (36.93%) medicine majors were included in the study. The average age of the sample in years was 20 (SD = 1.68).

### Differential Item Functioning Analyses

**Figure 1:**
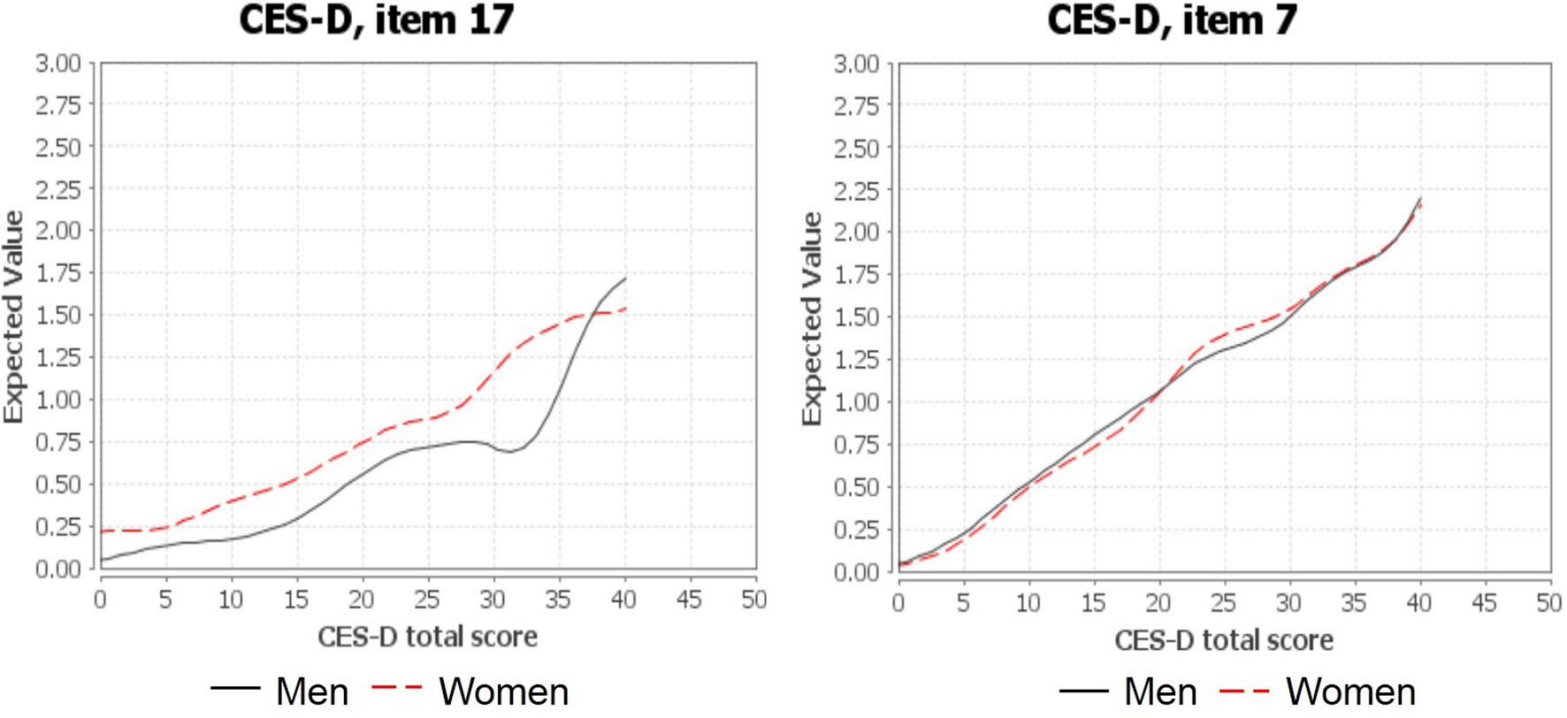
Item characteristic curves for items 17 and 7.

To verify that item 17 (“I had crying spells”) produced gender bias, differential item functioning analysis was conducted. As shown in **Figure 1**, the item characteristic curve of men differed markedly from the curve of women, suggesting that women were more likely to choose a higher response option than men. For the remaining items, their item characteristic curves demonstrated negligible difference between the male and female group. An example of item 7 was also presented in **Figure 1** for illustrative purposes.

### Psychometric Properties of the Chinese CES-D

**Figure 2:**
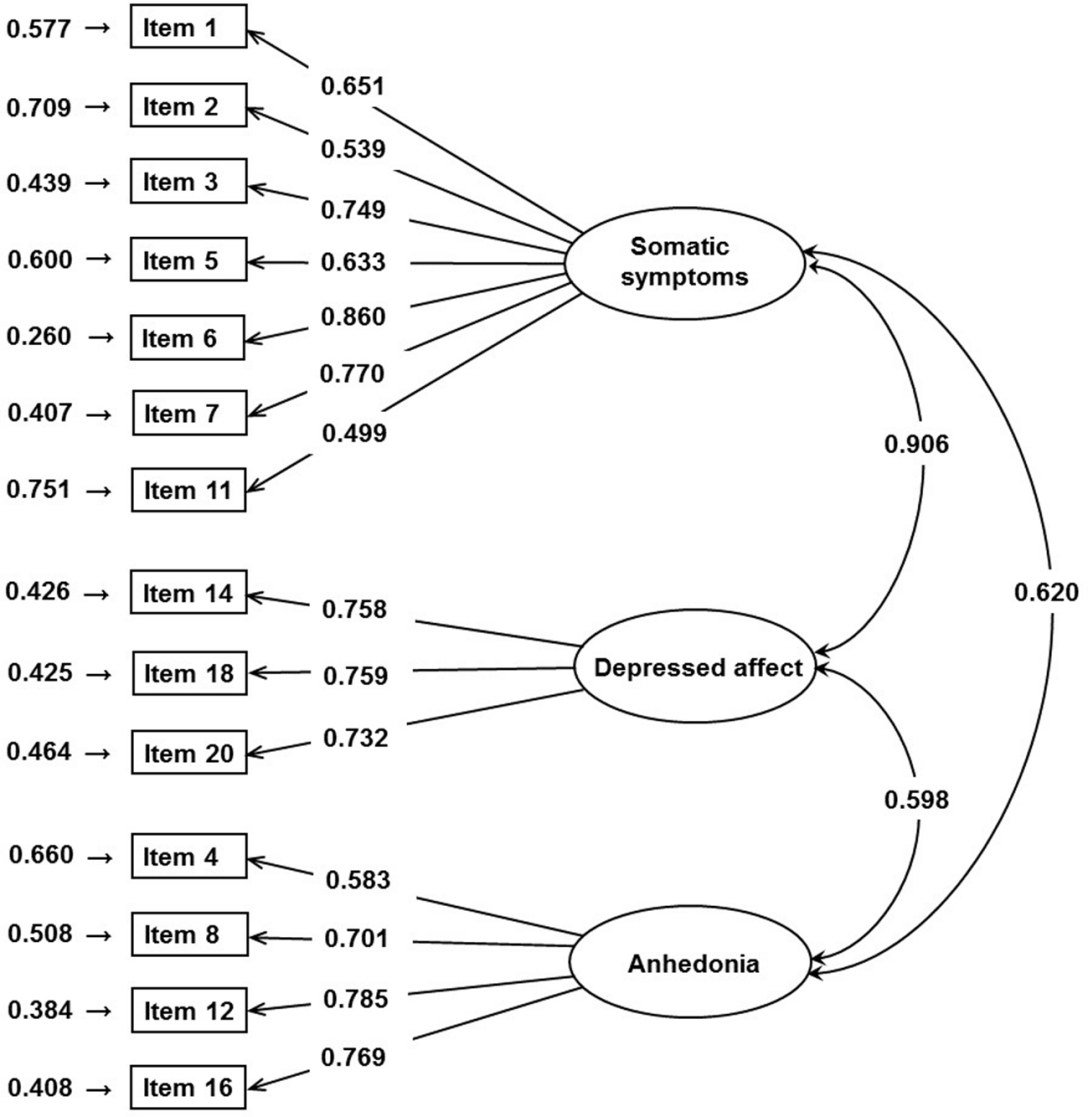
Path diagram of the revised exploratory factor analysis (EFA) model.

Each of the 1,920 participants was randomly assigned to Sample One or Sample Two. As a result, 963 and 957 participants were randomly assigned to Sample One and Sample Two, respectively. Results of independent *t* tests and chi-square tests indicated that the students in the two samples were not different with regard to CES-D total score (t = 1.068, *p* = 0.286), gender (χ^2^ = 1.536, *p* = 0.215), grade (χ^2^ = 0.002, *p* = 0.965), and major (χ^2^ = 2.524, *p* = 0.283). The results suggested that the random split assignment is appropriate. An initial EFA, including all 20 items, was conducted. A series of statistics indicated that EFA is appropriate for the current dataset, including Kaiser–Meyer–Olkin (KMO) = 0.939 and Bartlett’s *p* < 0.001. The EFA results on Sample One suggested extraction of three factors, which accounts for 52.68% of total variance. The three factors were somatic symptoms (item 1∼3, 5∼7, 11), negative affect (item 14, 15, 17∼20), and anhedonia (item 4, 8, 12, 16). Three of the 20 items, including items 9, 10, and 13, had weak factor loadings (<0.5), suggesting removal of the three items (23, 35). Pattern and structure coefficients are presented in **Table 1**. The model derived by EFA and five previously reported models were evaluated by CFA with and without the three items (item 15, 17, and 19). The fit indices are summarized in **Table 2**. Two of the seven models fit the data well, that is, the Carleton model and model derived by EFA but without the three items. Considering the AIC and BIC values as well as the difference in the AIC and BIC values, this suggests that the model derived by EFA but without the three items fit the data best. The CR values for the three factors were 0.855 (somatic symptoms), 0.794 (depressed affect), and 0.804 (anhedonia), suggesting satisfactory construct reliability. The AVE values were 0.465 (somatic symptoms), 0.562 (depressed affect), and 0.510 (anhedonia), suggesting acceptable convergent validity. The graphical expression of the path diagram of the revised EFA model was presented in **Figure 2**. The factor loadings for each item ranged from 0.499 to 0.860.

**Table 1:** Pattern and Structure Matrices.

CES-D Item	Pattern coefficients			Structure coefficients		
	Factor 1	Factor 2	Factor 3	Factor 1	Factor 2	Factor 3
18	0.77			0.80		0.51
19	0.73			0.79		0.51
17	0.73			0.66		
15	0.67			0.69		
14	0.61			0.68		
20	0.60			0.67		
**10**	**0.47**			0.66		0.59
**9**	**0.38**			0.64	0.54	0.61
16		0.80			0.81	
8		0.79			0.79	
12		0.73			0.76	
4		0.72			0.71	
5			0.84			0.74
7			0.74			0.80
6			0.60	0.58		0.76
11			0.58			0.58
1			0.57	0.51		0.67
2			0.53			0.54
3			0.50	0.57		0.68
**13**			**0.36**	0.53		0.55

Factor 1: Negative affect; Factor 2: Anhedonia; Factor 3: Somatic symptoms. CES-D, Center for Epidemiologic Studies Depression Scale.

**Table 2:** CFA fit indices and model comparisons for CES-D.

Model	Factors (items)	WLSMV χ^2^/*df*	TLI	CFI	RMSEA (90% CI)	AIC	BIC
Radloff et al. (11)	4 (20)	6.13	0.939	0.948	0.073 (0.069, 0.078)	38,926.006	39,247.017
Yen et al. (38)	3 (17)	5.84	0.948	0.955	0.071 (0.066, 0.076)	33,887.853	34,150.499
Lee et al. (39)	2 (19)	10.04	0.899	0.911	0.097 (0.093, 0.102)	37,373.738	37,655.839
Carleton et al. (12)	3 (14)	4.02	0.970	0.975	0.056 (0.050, 0.063)	28,365.536	28,584.407
Zhang et al. (18)	4 (20)	5.98	0.941	0.949	0.072 (0.068, 0.076)	38,905.708	39,226.719
Newly derived model	3 (17)	5.28	0.954	0.960	0.067 (0.062, 0.072)	33,534.185	33,796.830
The revised EFA model	3 (14)	3.74	0.972	0.978	0.053 (0.047, 0.060)	28,342.435	28,561.306

CFA, confirmatory factor analysis.

**Figure 3:**
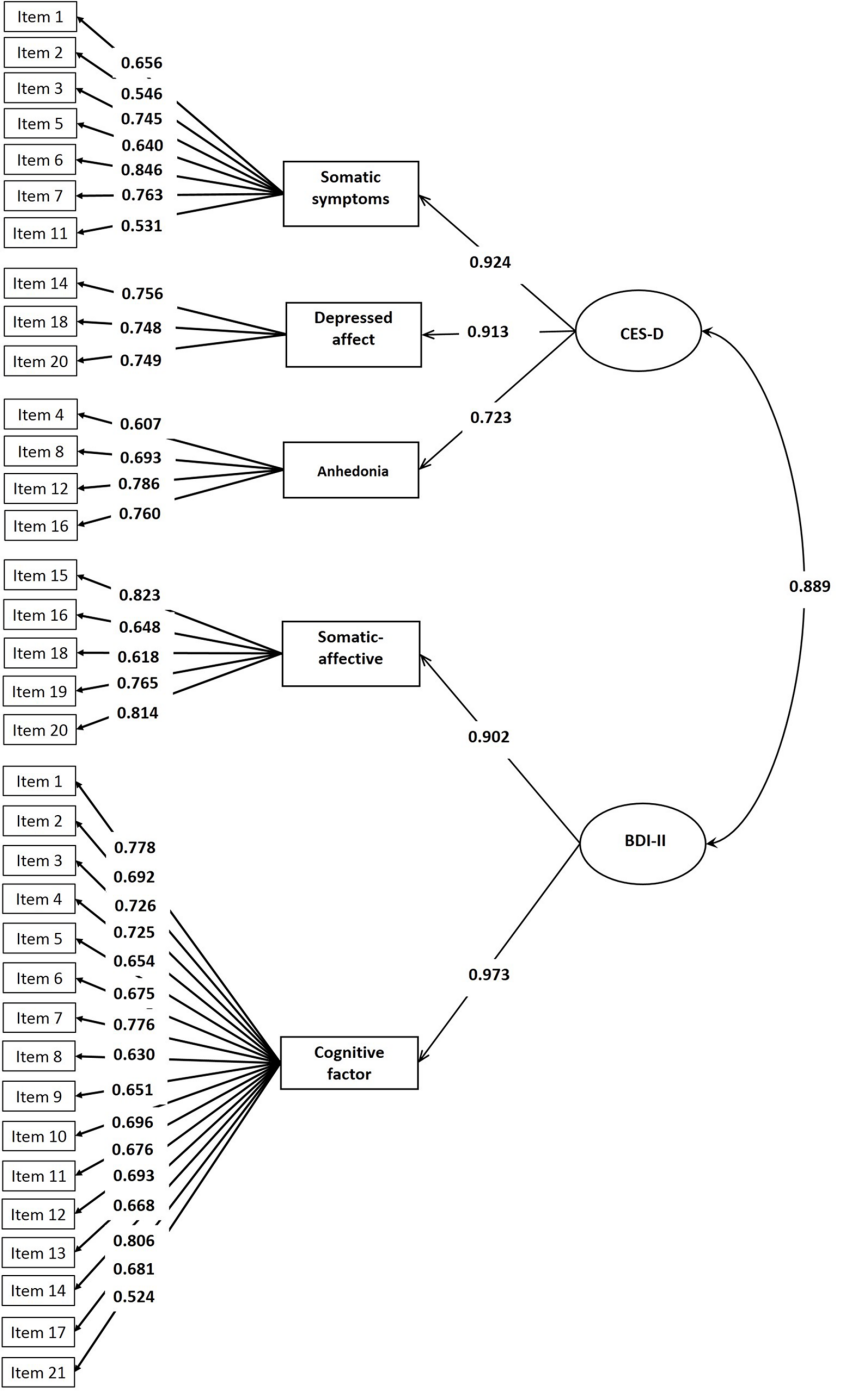
Measurement model for the subscales of CES-D and BDI-II.

The mean scores of the 14-item CES-D, BDI-II, positive affect, and negative affect were 12.38 (SD = 6.89), 9.80 (SD = 8.90), 27.39 (SD = 7.08), and 17.75 (SD = 6.18), respectively. Correlations between the 14-item CES-D, BDI-II, and PANAS are presented in **Table 3**. The Chinese CES-D scores were significantly correlated with the BDI-II (r = 0.74, P < 0.01), positive affect (r = −0.58, P < 0.01), and negative affect (r = 0.63, P < 0.01). We built a measurement model for the 14-item CES-D (somatic symptoms, negative affect and anhedonia) and BDI-II (somatic-affective and cognitive factor). The path diagram of this measurement model is presented in **Figure 3**. The factor loadings for CES-D items range from 0.531 to 0.846 and for CES-D based first-order factors range from 0.723 to 0.924. The factor loadings for BDI-II items range from 0.524 to 0.823 and for BDI-II based first-order factors range from 0.902 to 0.973. The measurement model produces a correlation of 0.889 between the CES-D and BDI-II based on second-order factor analysis. The two-factor model with correlated factors fits the data well (χ2 = 1,922.809, df = 554, RMSEA = 0.051, CFI = 0.937, TLI = 0.932).

**Table 3:** Correlations between the subscales of CES-D, BDI-II, and PANAS.

	CES-D-14 item	CES-D-20 item	BDI-II	CES-D1	CES-D2	CES-D3	BDI-II1	BDI-II2	PA	NA
CES-D-20	0.975**									
BDI-II	0.735**	0.744**								
CES-D1	0.909**	0.895**	0.679**							
CES-D2	0.788**	0.824**	0.613**	0.666**						
CES-D3	0.743**	0.674**	0.508**	0.456**	0.400**					
BDI-II1	0.632**	0.618**	0.845**	0.632**	0.487**	0.392**				
BDI-II2	0.719**	0.735**	0.975**	0.645**	0.615**	0.513**	0.720**			
PA	−0.578**	−0.537**	−0.479**	−0.452**	−0.368**	−0.596**	−0.422**	−0.464**		
NA	0.631**	0.667**	0.563**	0.586**	0.589**	0.388**	0.415**	0.578**	−0.236**	

CES-D-14 item, 14-item CES-D total score; CES-D-20 item, 20-item CES-D total score; BDI-II, BDI-II total score; CES-D1, Somatic symptoms; CES-D2, Depressed symptoms; CES-D3, Anhedonia; BDI-II1, Somatic-affective; BDI-II2, Cognitive factor; PA, Positive affect schedule total score; NA, Negative affect schedule total score. BDI-II, Beck Depression Inventory-II; PANAS, Positive and Negative Affect Schedule.

**P < 0.01.

The 14-item CES-D scale had satisfactory internal consistency with a Cronbach’s coefficient alpha of 0.87. The average inter-item correlation for all items was 0.32, which was acceptable and suggested that the items measure the same construct well. The average inter-item correlation with the reverse-scored items (item 4, 8, 12, and 16) and without the reverse-scored items was 0.45 and 0.37, respectively. The corrected item-total correlation coefficients for all items ranged from 0.400 (item 11) to 0.697 (item 6) (**Table 4**).

**Table 4:** Internal consistency of CES-D.

	Scale mean if item deleted	Corrected item-total correlation	Cronbach’s alpha if item deleted
1. I was bothered by things that usually don’t bother me.	11.56	0.533	0.857
2. I did not feel like eating; my appetite was poor.	11.77	0.402	0.863
3. I felt that I could not shake off the blues even with help from my family or friends.	11.70	0.608	0.852
4. I felt I was just as good as other people.	10.98	0.412	0.864
5. I had trouble keeping my mind on what I was doing.	11.23	0.502	0.858
6. I felt depressed.	11.43	0.697	0.847
7. I felt that everything I did was an effort.	11.55	0.648	0.850
8. I felt hopeful about the future.	11.25	0.465	0.860
11. My sleep was restless.	11.68	0.400	0.864
12. I was happy.	11.18	0.536	0.856
14. felt lonely.	11.48	0.556	0.855
16. I enjoyed life.	11.42	0.501	0.858
18. I felt sad.	11.65	0.578	0.854
20. I could not get “going.”	12.05	0.504	0.859

### Prevalence of Subthreshold Depression

**Figure 4:**
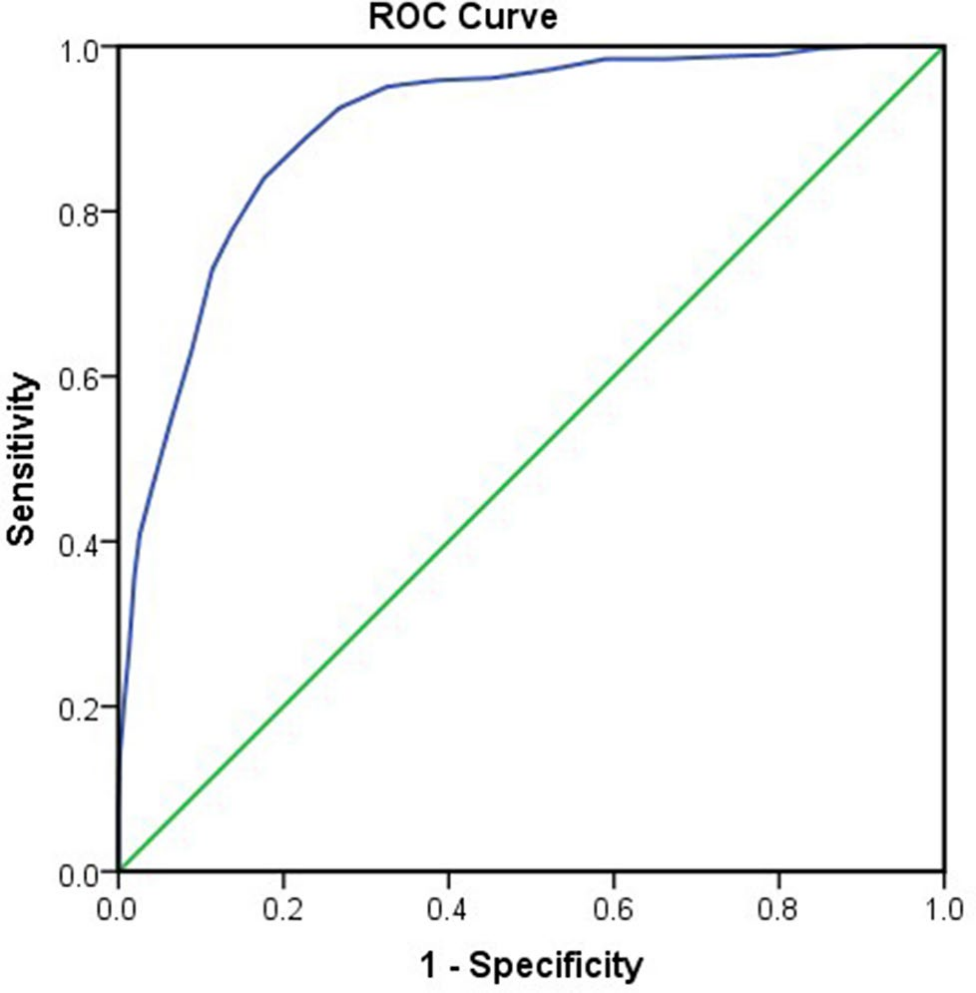
Receiver operator characteristic (ROC) curve analysis of the 14-item CES-D.

For the 20-item CES-D, the scores ranged from 0 to 57, and the average score was 16.03 (SD = 9.62). The prevalence of subthreshold depression was 32.7% considering the cut-off score of 20. For the 14-item CES-D, the scores ranged from 0 to 42 and the average score was 12.38 (SD = 6.89). The ROC curve of the 14-item CES-D is drawn in **Figure 4**, with an area under the curve (AUC) of 0.903 (95%CI: 0.887 to 0.919). The optimal cut-off point of 15.5 was determined by maximizing both the sensitivity (0.840) and specificity (0.824), with positive predictive values of 0.547 and negative predictive values of 0.953. The prevalence of subthreshold depression was 31% considering a cut-off value of 16. A multiple regression was calculated to predict the CES-D score based on demographic variables of gender, grade, and major. The results show that the model is not significant, F(4, 1,915) = 2.128, *P* = 0.075. This suggests that the model cannot significantly predict the CES-D score.

## Discussion

To our best knowledge, this is the first study to evaluate the psychometric properties of the Chinese version of the CES-D for assessing subthreshold depression in Chinese university students. The results indicate that the CES-D is a reliable and valid instrument for assessing subthreshold depression in Chinese university students. The fit statistics suggested that the newly derived model with 14 items provides the best fit for the current data. The prevalence of subthreshold depression is 32.7% for the 20-item CES-D and 31% for the 14-item CES-D, and there is no significant difference in the demographic correlates of gender, grade, and major.

### Psychometric Properties of the Chinese CES-D

Previous studies have highlighted that item 17 (“I had crying spells”) may have gender bias (12–13, 14, 44). As suggested by differential functioning analysis, the female group tended to respond higher compared with the male group. This finding is consistent with previous studies that reported that item 17 has gender bias (12, 13, 45). It is important to remove item 17 from the CES-D to achieve validation of a summary score to indicate the level of depression. In addition to item 17, the two interpersonal items (item 15, 19) may also be problematic. First, there is no theoretical support for including social items in an assessment of depression. The current DSM-V diagnosis manual (**Supplementary Material**) does not consider interpersonal problems a criteria to depression (15). In contrast, the two interpersonal items may assess symptoms of other disorders, such as social anxiety disorder (16, 46). Secondly, there are only two items for the interpersonal factor, which would result in psychometric difficulties (44, 47). Finally, a number of studies removed the two interpersonal items and produced a more validated measure of depression (12, 13). One study suggested that the two interpersonal items were unable to distinguish non-depressed and depressed patients with HIV/AIDS (48). Finally, in the original CES-D model developed by Radloff (1977) and other CES-D models (47, 49, 50), it is noted that the correlation between the interpersonal factor and other factors is very low. As a result, we evaluated the EFA models with and without the three items (item 15, 17, and 19).

The present study used confirmatory analysis to investigate factor structures of the Chinese CES-D, and the results suggested that the newly derived model with 14 items produced the best fit indices. The current results replicate the increasingly demonstrably robust results from Carleton et al. (12) with a Chinese sample and provide support for the three-factor structure of CES-D suggested by Carleton et al. (12). There is a small difference between the current model and the Carleton model. It was noted that items 3 (“blues”) and 6 (“felt depressed”) moved from the depressed affect to somatic symptoms, whereas item 20 (“could not get going”) moved from somatic symptoms to the depressed affect. It seems that the Chinese university students were confused about the difference between depressed affect and somatic symptoms. Numerous studies have shown that the Chinese tend to express somatic symptoms of depression, whereas people in Western countries tend to emphasize psychological symptoms of depression. For example, a study found that Chinese had higher endorsement rates for somatic symptoms compared with Euro-Australians, who in turn had higher endorsement rates for psychological symptoms (51). These differences would be lessened as Chinese-Australians adapt to mainstream Australian society (52). Similarly, another study also found that the Chinese reported more somatic symptoms than the Euro-Canadians (53). However, several studies have failed to find support for the relationship between culture and symptom expression. For instance, using the CES-D, Yen et al. (38) found that a Chinese student sample reported a significantly lower level of somatic depressive symptom endorsement compared with an American student sample (38). Importantly, several studies highlighted the role that cultural norms play in symptom expression. Drawing from a social identity perspective, a study found that increased somatic symptom expression occurred only when Asian participants were willing to endorse collectivism norms and identified strongly with Asian culture (54).

In the general populations, the CES-D has exhibited a good internal consistency with Cronbach’s alpha coefficients ranging from 0.83 to 0.95 (55–57). The CES-D also showed good reliability, with a Cronbach’s alpha from 0.89 to 0.92 in university students from Japan, US, and Taiwan (58–60). Furthermore, the Chinese version of CES-D has shown satisfactory reliability in children, American Chinese women, community residents, and elderly in Hong Kong, with Cronbach’s alpha values of 0.82, 0.86, 0.86, and 0.90 (17, 61–63). In the present study, the Cronbach’s alpha reached 0.87, indicating a good reliability when used in university students. The BDI-II and PANAS were used to evaluate the criterion validity of the CES-D. The results showed that the CES-D scores were positively correlated with BDI-II and negative affect scores and negatively correlated with positive affect scores, demonstrating a good criterion validity of the Chinese CES-D in university students. Furthermore, the two-factor measurement model fits well with the data, suggesting that there is an overlap in the constructs underlying the subscales of the CES-D and BDI-II. This is consistent with the correlation between total scores of the two scales and the theory on depression.

Prevalence of subthreshold depression among the Chinese university students.

The prevalence of subthreshold depression, as assessed by the 20-item CES-D and 14-item CES-D, in university students was 32.7% and 31%, respectively. This is slightly higher than the 23.8% and 30.39% incidence reported in two meta-analysis studies on Chinese university students (64, 65). This is reasonable since the majority of studies used diverse measures, such as the Self-rating Depression Scale (66), BDI (67), and Hamilton Depression Scale (68), which evaluated depression rather than subthreshold depression. In addition, we found no significant differences in subthreshold depression with regard to gender, grade and major. This is consistent with previous studies suggesting no significant differences in depressive symptoms between male and female students (69–71). This may be because Chinese female university students are equal as their male peers in many ways, such as political rights, job opportunities, and pressure from academics and life. Regarding grade, previous studies have shown inconsistent findings (71, 72) that may be explained by different measurement tools and sample errors.

### Limitations

First, we did not have a diagnostic instrument for depression diagnosis, although a number of excellent diagnostic instruments exist for depression diagnosis, such as Diagnostic Interview Schedule (DIS) and Structured Clinical Interview for DSM Disorder (SCID). However, using such instruments is time consuming and unfeasible in population-based surveys. As a result, we lack a gold standard for depression diagnosis to investigate sensitivity, specificity, positive and negative predictive values of the Chinese CES-D to predict depression or subthreshold depression. Second, the sample was recruited from only two universities in Guangzhou, which limited the generalization of the results of subthreshold depression prevalence to a larger university population in China. Third, the sample was not followed up and test-retest reliability could not be examined. Finally, we only investigated the university student sample. Construct and external validity should be investigated in clinical samples.

## Conclusions

The present findings indicate that the three-factor structure with 14 items of CES-D has satisfactory psychometric properties as an instrument for assessing subthreshold depressive symptoms in Chinese university students. The prevalence of subthreshold depression reaches 32.7% for the 20-item CES-D and 31% for the 14-item CES-D, and there is no significant difference in the variables of gender, grade, and major.

## Data Availability Statement

All datasets generated for this study are included in the manuscript and the supplementary files.

## Ethics Statement

This study was carried out in accordance with the recommendations of Declaration of Helsinki with written informed consent from all subjects. All subjects gave written informed consent in accordance with the Declaration of Helsinki. The protocol was approved by the Ethics Committee of School of Medical Science at Jinan University, China.

## Author Contributions

LJ contributed to data collection, data analysis, discussion on results, writing, and preparation of the manuscript. YW contributed to study design, data analyses, writing and preparation of the manuscript. YZ, RL, HW, CL, and YLW contributed to data collection, data analyses, and discussion on results. QT contributed to study design, data collection, results interpretation, discussion of results, writing, and preparation of the manuscript. All authors read and approved the final manuscript.

## Funding

This work was supported by research grants from the National Natural Science Foundation of China (81601969, 81671670, 81501456, and 81471650); Science and Technology Program of Guangdong (2018B030334001); Fundamental Research Funds for the Central Universities; Planned Science and Technology Project of Guangdong Province, China (2014B020212022); and Planned Science and Technology Project of Guangzhou, China (201508020004, 20160402007, and 201604020184).

## Conflict of Interest Statement

The authors declare that the research was conducted in the absence of any commercial or financial relationships that could be construed as a potential conflict of interest.
