# Supplementary material for: The Reliability and Validity of the Center for Epidemiologic Studies Depression Scale (CES-D) for Chinese University Students
Source: Front Psychiatry. 2019 May 21;10:315. doi: 10.3389/fpsyt.2019.00315 (PMC6537885; doi:10.3389/fpsyt.2019.00315)
Supplement: Supplementary file 1 [file Table_1.docx]

CES-D

|  | None of the time (＜1 day) | Little of the time  (1-2 days) | Some of the time  (3-4 days) | Most of the time  (5-7 days) |
| --- | --- | --- | --- | --- |
| 1 I was bothered by some small things that usually do not bother me. | 1 | 2 | 3 | 4 |
| 2 I didn't want to eat and my appetite was poor. | 1 | 2 | 3 | 4 |
| 3 I could not shake off the blues even with help from my family and friends. | 1 | 2 | 3 | 4 |
| 4 I felt as good as other people. I feel as good as the average person. | 1 | 2 | 3 | 4 |
| 5 It was difficult for me to concentrate on what I was doing. | 1 | 2 | 3 | 4 |
| 6 I felt depressed. | 1 | 2 | 3 | 4 |
| 7 I felt that everything I did was an effort. | 1 | 2 | 3 | 4 |
| 8 I felt that the future is promising. | 1 | 2 | 3 | 4 |
| 9 I felt that my life is a failure. | 1 | 2 | 3 | 4 |
| 10 I felt fearful. | 1 | 2 | 3 | 4 |
| 11 My sleep was not good. | 1 | 2 | 3 | 4 |
| 12 I was happy. | 1 | 2 | 3 | 4 |
| 13 I talked less than usual. | 1 | 2 | 3 | 4 |
| 14 I felt lonely. | 1 | 2 | 3 | 4 |
| 15 I think people are not friendly to me. | 1 | 2 | 3 | 4 |
| 16 I enjoyed life. | 1 | 2 | 3 | 4 |
| 17 I used to cry. | 1 | 2 | 3 | 4 |
| 18 I felt sad. | 1 | 2 | 3 | 4 |
| 19 I felt that people don't like me. | 1 | 2 | 3 | 4 |
| 20 I felt that I could not continue my daily work. | 1 | 2 | 3 | 4 |
