# Supplementary material for: The Reliability and Validity of the Center for Epidemiologic Studies Depression Scale (CES-D) for Chinese University Students
Source: Front Psychiatry. 2019 May 21;10:315. doi: 10.3389/fpsyt.2019.00315 (PMC6537885; doi:10.3389/fpsyt.2019.00315)
Supplement: Supplementary file 2 [file Table_2.pdf]

Major Depressive Disorder

Diagnostic Criteria

A. Five (or more) of the following symptoms have been present during the same 2-week period and represent a change from previous functioning; at least one of the symptoms is either (1) depressed mood or (2) loss of interest or pleasure.

Note: Do not include symptoms that are clearly attributable to another medical condition.

1. Depressed mood most of the day, nearly every day, as indicated by either subjective report (e.g., feels sad, empty, hopeless) or observation made by others (e.g., appears tearful). (**Note:** In children and adolescents, can be irritable mood.)

2. Markedly diminished interest or pleasure in all, or almost all, activities most of the day, nearly every day (as indicated by either subjective account or observation).

3. Significant weight loss when not dieting or weight gain (e.g., a change of more than 5% of body weight in a month), or decrease or increase in appetite nearly every day.

(**Note:** In children, consider failure to make expected weight gain.)

4. Insomnia or hypersomnia nearly every day.

5. Psychomotor agitation or retardation nearly every day (observable by others, not merely subjective feelings of restlessness or being slowed down).

6. Fatigue or loss of energy nearly every day.

7. Feelings of worthlessness or excessive or inappropriate guilt (which may be delusional) nearly every day (not merely self-reproach or guilt about being sick).

8. Diminished ability to think or concentrate, or indecisiveness, nearly every day (either by subjective account or as observed by others).

9. Recurrent thoughts of death (not just fear of dying), recurrent suicidal ideation without a specific plan, or a suicide attempt or a specific plan for committing suicide.

B. The symptoms cause clinically significant distress or impairment in social, occupational, or other important areas of functioning.

C. The episode is not attributable to the physiological effects of a substance or to another medical condition.

Note: Criteria A–C represent a major depressive episode.

Note: Responses to a significant loss (e.g., bereavement, financial ruin, losses from a natural disaster, a serious medical illness or disability) may include the feelings of intense sadness, rumination about the loss, insomnia, poor appetite, and weight loss noted in Criterion A, which may resemble a depressive episode. Although such symptoms may be understandable or considered appropriate to the loss, the presence of a major depressive episode in addition to the normal response to a significant loss should also be carefully considered. This decision inevitably requires the exercise of clinical judgment based on the individual's history and the cultural norms for the expression of distress in the context of loss.1

D. The occurrence of the major depressive episode is not better explained by schizoaffective disorder, schizophrenia, schizophreniform disorder, delusional disorder, or other specified and unspecified schizophrenia spectrum and other psychotic disorders.

E. There has never been a manic episode or a hypomanic episode.

Note: This exclusion does not apply if all of the manic-like or hypomanic-like episodes are substance-induced or are attributable to the physiological effects of another medical condition.

----

1 In distinguishing grief from a major depressive episode (MDE), it is useful to consider that in grief the predominant affect is feelings of emptiness and loss, while in MDE it is persistent depressed mood and the inability to anticipate happiness or pleasure. The dysphoria in grief is likely to decrease in intensity over days to weeks and occurs in waves, the so-called pangs of grief. These waves tend to be associated with thoughts or reminders of the deceased. The depressed mood of MDE is more persistent and not tied to specific thoughts or preoccupations. The pain of grief may be accompanied by positive emotions and humor that are uncharacteristic of the pervasive unhappiness and misery characteristic of MDE. The thought content associated with grief generally features a preoccupation with thoughts and memories of the deceased, rather than the self-critical or pessimistic ruminations seen in MDE. In grief, self-esteem is generally preserved, whereas in MDE feelings of worthlessness and self-loathing are common. If self derogatory ideation is present in grief, it typically involves perceived failings vis-à-vis the deceased (e.g., not visiting frequently enough, not telling the deceased how much he or she was loved). If a bereaved individual thinks about death and dying, such thoughts are generally focused on the deceased and possibly about "joining" the deceased, whereas in MDE such thoughts are focused on ending one's own life because of feeling worthless, undeserving of life, or unable to cope with the pain of depression.
